# AI-Powered Precision: Revolutionizing Atrial Fibrillation Detection with Electrocardiograms

**DOI:** 10.3390/jcm14144924

**Published:** 2025-07-11

**Authors:** Ameen Nasser, Mateusz Michalczak, Anna Żądło, Tomasz Tokarek

**Affiliations:** 1 Center for Innovative Medical Education, Jagiellonian University Medical College, 30-688 Krakow, Poland; atnasser96@gmail.com (A.N.); matibielsko@gmail.com (M.M.); anna.zadlo@uj.edu.pl (A.Ż.); 2 Center for Invasive Cardiology, Electrotherapy and Angiology, 33-300 Nowy Sacz, Poland

**Keywords:** artificial intelligence, atrial fibrillation, electrocardiography, detection, AI-powered ECG

## Abstract

Atrial fibrillation (AF) is a common cardiac arrhythmia linked to an increased risk of stroke, heart failure, and mortality, yet its diagnosis remains challenging due to its intermittent and often asymptomatic nature. Traditional methods, such as standard electrocardiography (ECG) and prolonged cardiac monitoring, have limitations in terms of cost, accessibility, and diagnostic yield. Artificial intelligence (AI), particularly machine learning (ML) and deep learning, has emerged as a promising tool for AF detection and prediction by analyzing ECG data with high accuracy. AI models can identify subtle patterns in ECG signals that may indicate AF, even when the arrhythmia is not actively present, improving early diagnosis and risk stratification. Additionally, AI-powered ECG analysis has been integrated into wearable and mobile health devices, expanding screening capabilities beyond clinical settings. While studies have demonstrated AI’s effectiveness, challenges such as data bias, model reliability across diverse populations, and regulatory considerations must be addressed before widespread clinical adoption. If these obstacles are overcome, AI has the potential to revolutionize AF management by enabling earlier detection, reducing the need for resource-intensive monitoring, and improving patient outcomes.

## 1. Introduction

The prevalence of atrial fibrillation (AF) is rising due to aging populations and an increase in risk factors such as hypertension, diabetes, and obesity. In 2021, AF became the most prevalent arrhythmia worldwide, affecting 52.55 million people. In the United States alone, an estimated 3–6 million individuals have AF, with projections indicating a rise to 6–16 million by 2050. Similarly, in Europe, the prevalence was approximately 9 million in 2010 and is expected to reach 14 million by 2060 [1,2].

AF is a major contributor to stroke, heart failure, and overall cardiovascular mortality, making its timely detection crucial for effective management. However, AF diagnosis remains challenging due to its often asymptomatic and paroxysmal nature. Many patients experience intermittent episodes that may not be captured during routine clinical evaluations, leading to underdiagnosis and delayed intervention [1]. AF detection primarily relies on electrocardiography (ECG) using 12-lead recordings, Holter monitors, and implantable loop recorders. While effective, these approaches have significant limitations. In patients with paroxysmal atrial fibrillation, a single ECG taken during a routine clinical visit detects AF before a thromboembolic event in 64% of cases. Consequently, there is an increasing need for more efficient, accessible, and cost-effective tools for AF screening and diagnosis [3].

Artificial Intelligence (AI) holds promise in cardiology, particularly for already-manifested AF, allowing for efficient detection and prediction. By leveraging machine learning (ML) and deep learning techniques, AI can analyze large data sets of ECG signals to detect patterns that may be imperceptible to human clinicians. AI has been developing at a rapid pace in the field of cardiology to enhance efficiency (refer to Section 1.1) [4,5]. AI models can process both 12-lead ECGs and single-lead wearable device recordings with high accuracy, allowing for the more reliable identification of AF. Moreover, AI-powered ECG analysis can extend beyond detection to prediction, identifying individuals at high risk of developing AF even before the onset of AF/thrombotic episodes. This predictive capability is especially valuable for guiding early interventions in high-risk populations, such as those with a history of stroke of unknown origin [5]

Despite its promise, the integration of AI into AF detection and management presents challenges. AI models require extensive training on diverse datasets to ensure they perform reliably across different patient populations. Issues related to algorithmic bias, clinical validation, and regulatory approval must be addressed before AI-driven AF detection can be widely implemented in healthcare settings. Additionally, ensuring AI interpretability and gaining clinician and patient trust are essential for successful adoption [5].

### 1.1. Historical Perspectives

The development of AI in AF detection and monitoring has progressed significantly over the past decade (see Table 1). Initially limited in scope, AI applications advanced rapidly with the introduction of deep learning techniques that enabled the accurate identification of AF from standard electrocardiograms, even during sinus rhythm. This breakthrough improved early and asymptomatic AF detection. The integration of AI into wearable devices further enhanced real-time, continuous heart rhythm monitoring, allowing for large-scale population screening. These technologies demonstrated high predictive value and opened up new possibilities for timely and accessible AF diagnosis outside traditional clinical settings [4]. Soon after, multiple models and very large datasets began to be integrated with AI, further enhancing detection and monitoring.

### 1.2. Mechanisms and Pathophysiology of AF

To understand how AI can detect AF, the pathophysiology of AF must be discussed. AF is a complex heart arrhythmia that results from structural and electrical abnormalities in the atria, leading to disorganized and rapid electrical impulses [1]. Several factors contribute to the generation of these chaotic impulses:

- Focal and ectopic impulse generation: disorganized electrical impulses can originate from ectopic sites, most commonly within the pulmonary veins, initiating AF episodes. These foci are composed of cardiomyocytes that arise during development, since the pulmonary vein initially forms as an outgrowth from the primitive left atrium.
- Electrical reentry currents: under normal conditions, electrical impulses travel in an organized manner across the atria. However, once AF is triggered, abnormal electrical impulses are sustained by reentrant circuits that propagate chaotically due to shortened action potentials and refractory periods. Such reentrant currents are described to be spiral in nature, causing widespread contractions throughout the atria.
- Electrical remodeling: sustained AF allows the remodeling of electrical pathways in the atria. Remodeling includes a reduction in L-type calcium channels, which lead to a shorter action potential and refractory periods. There is also an increase in inward potassium currents, which enhances repolarization, further reducing the refractory period. Additionally, the downregulation of sodium channels slows conduction velocity and increases the probability of a reentrant current [1].

Hence, AF is a multifactorial arrhythmia driven by various interacting mechanisms. These factors create a self-perpetuating cycle of unorganized atrial contractions, making the condition progressively harder to manage. Detecting these abnormal electrical patterns is crucial for effective AF diagnosis and treatment. ECG is commonly used to identify such disruptions, and AI can enhance ECG interpretation, improving both predictive power and diagnostic accuracy [3].

### 1.3. AI in AF Detection and Prediction

In AF research, the term detection refers to the identification of AF during an active episode using current ECG or Photoplethysmography (PPG) recordings, providing immediate diagnostic value. In contrast, prediction focuses on assessing a patient’s future risk of developing AF, often through the analysis of ECG data recorded during sinus rhythm. This distinction is important: detection informs real-time clinical decisions, while prediction supports early interventions and long-term management strategies [6].

AI detects AF on ECGs by analyzing both traditional electrophysiological markers and more complex, subclinical patterns. It evaluates irregular R–R intervals and the absence of consistent P-waves, which are hallmark signs of AF [1,2]. AI algorithms, particularly those using deep learning, can also identify subtle features such as low-amplitude fibrillatory waves, changes in T-wave morphology, and hidden signatures during sinus rhythms that may indicate future AF episodes. Moreover, AI employs nonlinear metrics such as entropy and other mathematical descriptors of signal complexity to differentiate AF from other arrhythmias or noise [3].

Accurate atrial fibrillation detection, particularly with wearable and mobile devices, relies heavily on preprocessing methods that minimize the effects of noise and motion artifacts, which can compromise signal integrity. Bandpass filtering (commonly 0.5–40 Hz for ECG and 0.5–8 Hz for PPG) is routinely applied to eliminate baseline drift and high-frequency interference [7,8,9]. Adaptive thresholding further enhances detection by adjusting sensitivity levels in response to changing signal conditions, ensuring the reliable identification of cardiac features even in the presence of noise [8]. To safeguard analysis quality, signal quality assessment (SQA) techniques—such as evaluating kurtosis, power ratios, or template correlations—are used to detect and exclude segments with poor signal quality [7,10,11]. Additionally, advanced signal processing strategies, including independent component analysis, empirical mode decomposition, and motion artifact correction informed by accelerometer data, are increasingly integrated into AI pipelines to strengthen performance under real-world conditions [9,11]. These preprocessing measures are vital to providing AI models with clean, high-quality input data, which in turn supports accurate and generalizable AF detection across varied settings and patient populations.

One of the most promising techniques that allows AI to detect AF involves the application of convolutional neural networks (CNNs) to ECGs. This approach uses conventional ECG recordings, typically lasting 10 to 12 s, to detect potential AF occurrences, which enables the identification of AF-related patterns even when the heart is in normal sinus rhythm. The prediction of AF during apparent normal sinus rhythm potentially allows physicians to predict and reduce the risk of future episodes through treatment optimization [6]. While many models (see Table 2) depend on CNNs, there have also been recent developments in other models that either incorporate CNNs in their algorithm or constitute a completely new model.

## 2. Advancements in and Advantages of Using AI for AF Detection

Considerable progress has been made in the integration of AI to detect and predict AF. Deep learning algorithms have significantly improved the efficiency of AI in analyzing ECG data when compared to traditional diagnostic methods. For instance, Ahmad et al. [6] demonstrated that, when applied to 12-lead ECGs via CNNs, AI could detect the risk of AF based on the history of subtle changes in sinus rhythm. Originally designed for identifying silent AF in the general population, this artificial intelligence electrocardiogram (AI-ECG) tool holds promise for identifying individuals at high risk of AF or for detecting cases that may have gone undiagnosed in specific individuals. Research by Christopoulos et al. [7] further supported the effectiveness of this approach, showing that AI-ECG algorithms could successfully predict AF development in individuals with no prior history of the condition.

**Figure 1:**
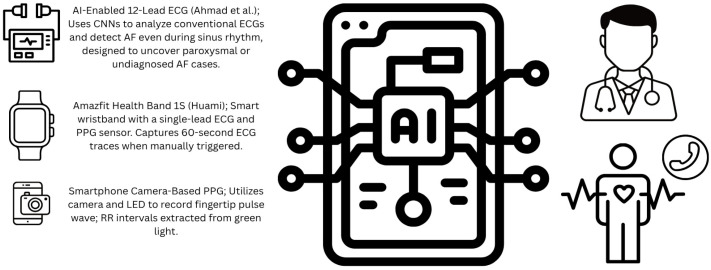
Summary of devices that use AI to detect AF. (Figure made using www.canva.com).

Wearable and handheld technologies (see Figure 1), including smartwatches and devices such as the Amazfit Health Band 1S (Huami Technology, Anhui, China)—a wrist-worn device featuring a single-channel ECG recorder alongside a high-precision PPG optical sensor—are becoming increasingly important in the detection of AF. The single-lead ECG function records a 60 s trace whenever the device is activated. When worn on the left wrist and with the right hand placed on the outer side of the ring, the reading reflects limb lead I. These AI-driven wearables enable continuous monitoring, enhancing AF detection accuracy by reducing both false positives and negatives. Although detecting asymptomatic AF continues to pose a challenge in terms of unpredictable silent episodes and anticoagulation treatment, cost-effective wearable solutions are making long-term screening viable for both healthy individuals and those at elevated risk [10]. To obtain a signal using smartphones, the camera lens and light emitting diode (LED) were aligned with the index fingertip. A five-minute video was recorded, with the pulse wave extracted from the green spectrum of the light signal. R–R intervals were automatically detected [13]. A systematic review and meta-analysis examined the diagnostic accuracy of smartphone-camera-based PPG applications compared to standard ECG for AF detection. This analysis encompassed 28 studies with a total of 11,404 participants, of whom 2950 had AF. The combined sensitivity was 94% (95% CI 92% to 95%) and the specificity was 97% (95% CI 96% to 98%), demonstrating that smartphone-based PPG technology can reliably identify AF with high accuracy; however, it also faces some limitations such as poor peripheral perfusion, motion artifacts, and tremor [11].

Moreover, AI models (see Table 2) have proven effective in analyzing large datasets to improve the accuracy of AF detection. These models have been trained to recognize specific probability thresholds during internal validation and apply them in test datasets, significantly improving AF detection. ML also aids in the identification of key risk factors for AF, such as age, hypertension, and obesity, which can assist in early diagnosis. AI’s ability to identify previously undetected AF in individuals with cryptogenic stroke is another important development [11]. Attia et al. [8] emphasized the potential for advanced computational models and large datasets to enhance the diagnosis and treatment of AF. The study included every Mayo Clinic ECG recording for patients above 18 between 31 December 1993 and 21 July 2017, accounting for 180,922 patients with 649,931 ECGs. Out of that data a set of 126,526 patients with 454,789 ECGs were used to train the algorithm/AI for detection and 64,340 ECGs from 18,116 patients were used for an internal validation test. By detecting minor abnormalities such as subtle differences between P-wave amplitudes and very miniscule R–R wave variability, the model successfully identified 3051 patients with confirmed AF.

AI can identify individuals at high risk of developing AF based on sinus rhythm ECGs, reducing risks and preventing complications in the future. Compared to conventional diagnostic methods, AI models typically demonstrate superior sensitivity and specificity, thus improving diagnostic accuracy [6]. Real-time monitoring through AI also facilitates quicker medical interventions, making it easier to manage AF cases promptly.

An Artificial-Intelligence-enabled electrocardiogram model has demonstrated effectiveness in identifying silent AF among patients with cryptogenic stroke. By analyzing ECGs recorded during sinus rhythm, the AI-ECG was able to differentiate cryptogenic stroke patients from those with established stroke causes [14]. A case report illustrated the utility of AI-ECG in a patient with recurrent cryptogenic stroke. The patient’s repeated ECGs and cardiac monitoring showed sinus rhythm; a retrospective AI-ECG analysis identified a high likelihood of AF risk as early as 12 years before the patient’s first thromboembolic event. This case highlights the potential of AI-ECG in early AF risk detection, reinforcing the importance of proactive monitoring and preventive strategies for high-risk individuals and influencing clinical decisions in cryptogenic stroke scenarios, raising the question of how often such high-risk patterns can be detected in otherwise healthy human beings [15]. An assessment of an AI model designed to detect previously undiagnosed paroxysmal AF in cryptogenic stroke patients revealed strong diagnostic accuracy using sinus rhythm ECGs. The model achieved an area under the curve (AUC) of 0.806, which improved to 0.880 with the addition of clinical variables. These findings indicate that AI-driven detection may significantly enhance patient outcomes by facilitating early intervention and improving secondary prevention strategies for ischemic stroke in cryptogenic stroke patients [16]. In a study by Wu et al. [10], the Stacking Ensemble Learning Method achieved high classification accuracy, with 92% overall accuracy, 88% sensitivity, and 96% positive predictive value. Given its high sensitivity, this method is recommended for large-scale AF screening, effectively identifying up to 88% of AF cases while distinguishing healthy individuals with 96% accuracy. However, performance in real-world settings may vary depending on population characteristics and ECG acquisition quality. Moreover, AI-driven models may potentially reduce the need for frequent hospital visits, lowering healthcare costs while enabling personalized management for AF patients. These models can tailor treatment strategies based on an individual’s specific risk profile, ensuring more targeted interventions [10].

Despite the progress that has been made, diagnosing paroxysmal AF remains challenging, as shown by the statistical performance results (refer to Section 4). The rhythm can closely resemble a normal sinus rhythm. Ensemble learning techniques, which train multiple models simultaneously, offer solutions to this issue, improving diagnostic performance. Techniques such as Bagging, AdaBoost, and Stacking have all shown promise in enhancing the accuracy of AF detection [17,18].

## 3. Challenges and Disadvantages of Using AI in AF Detection

One major concern with AI-based systems is the possibility of misdiagnosis, which can lead to unnecessary anxiety; conversely, missed AF detection can occur due to false positives or negatives. Dupulthys et al. [12] identified two key limitations of AI models in ECG-AI tools. First, these tools have yet to assess the AF burden, which is critical for understanding the impact of AF on patients and treatment decisions. Second, while guidelines suggest 30 s ECG recordings, the study used only 10 s recordings, casting doubt on the accuracy of the predictions. Another limitation is ECG quality variability, which can reduce diagnostic accuracy. Gahungu et al. [19] pointed out that including low-quality ECGs in the dataset could contribute to lower specificity, as the AI model may misclassify non-AF arrhythmias, sinus pauses, and ectopic beats commonly seen in patients with obstructive sleep apnea, which is a known cause of AF [20,21]. It is worth noting that sleep apnea itself is a known risk factor for AF [22]. The implementation of AI in clinical practice also requires significant financial investment and access to high-quality, diverse datasets. While some medical professionals are hesitant to rely solely on AI diagnoses, there are also concerns about data privacy, informed consent, and liability in cases of misdiagnosis [20].

Despite recent advancements, AI models face challenges in ensuring their effectiveness across diverse populations. AI algorithms trained on non-representative datasets may struggle to perform reliably in different groups (see Section 5), which could lead to misdiagnosis through false positives or negatives. While accessible, wearable devices including smartphones and smartwatches that use PPG sensors are prone to inaccuracies due to factors such as signal quality, tissue perfusion, movement, lighting conditions, and sensor placement [23]. A deep learning model called ArNet2 was developed to detect AF from long-term ECG recordings. Researchers tested how well the model worked across different groups, including various ethnicities, age ranges, and sexes. While ArNet2 performed well overall, there were noticeable differences in accuracy among demographic groups. These differences were mainly linked to a higher rate of atrial flutter in certain populations, indicating the limitations that can arise when training data are not fully representative [24]. Choosing the right evaluation metrics is especially important when assessing AI tools for AF detection—particularly with imbalanced datasets where AF events are relatively rare. Common metrics such as accuracy and AUC might not tell the whole story in these cases. To better understand model performance, it is helpful to include metrics such as positive predictive value (PPV), sensitivity, specificity, the F1 score, and precision–recall curves [5].

To build more reliable and consistent AI-based methods for detecting AF, it is essential to have well-labeled, standardized datasets and to validate these models through prospective clinical trials. Wearable technologies, such as patch monitors and smartwatches, are also making a significant impact—they provide a cost-effective, noninvasive way to continuously screen for AF. This is especially useful for high-risk groups, e.g., people recovering from surgery or those already diagnosed with AF. These AI-enabled wearables allow for long-term, passive monitoring, offering a convenient solution for managing AF. Still, more validation studies are needed. Looking ahead, research should focus on standardizing study methods, ensuring proper data labeling, and protecting patient privacy [25].

AI training requires access to large-scale clinical datasets, which raises concerns about data privacy and compliance with legal regulations. There is also skepticism among healthcare professionals regarding the reliance on AI for clinical decision making, which could impede the widespread adoption of AI-driven tools [26]. Strict regulatory approval and ethical considerations, particularly regarding AI’s role in critical medical decisions, represent significant challenges. Combining AI-generated interpretations with evaluations from trained healthcare professionals can help lower the risks that arise when relying too heavily on AI alone for diagnoses. Studies show that, while AI-based ECG analysis can provide helpful insights, current computer-generated readings still have their limitations. This is why expert review is so important: it adds a critical layer of oversight to help avoid potential misdiagnoses. Thanks to their wide availability and affordability, these AI tools are also well-suited for large-scale screening efforts [27].

## 4. Comparative Analysis

Most of the models reviewed in this study demonstrate high sensitivity (up to 98.1%), which reflects their ability to correctly detect true positive cases; this is important for identifying actual patients. Specificity, reaching 98.1%, indicates how well the models avoid false positives, ensuring that healthy individuals are not misdiagnosed. Accuracy, reported to be as high as 96.32%, measures the overall correctness of the model across both positive and negative predictions. While there is no universally agreed benchmark for sensitivity, specificity, or accuracy in cardiovascular AI diagnostics, most of the studies reviewed report values exceeding 80% (see Table 3). This suggests that a performance threshold of around 80% is generally considered acceptable in this domain.

The strongest performances came from models using deep learning CNNs and PPG-AI algorithms, all showing sensitivity and specificity above 94% (excluding the male readings in Biton et al. [24]), indicating reliable detection and low false alarms. Smartphone-based and wearable approaches also showed promise, though accuracy varied more widely.

Some models used in different studies showed lower sensitivity, specificity and accuracy than others; such discrepancies can be attributed to variability in the methods of each study, such as demographics, tremors, poor peripheral perfusion, and other uncontrolled variables that might affect ECG recording.

## 5. Demographic Analysis

Demographic analyses play a crucial role in evaluating the effectiveness and generalizability of AI models designed to detect AF across diverse population subgroups. Variations in age, sex, ethnicity, and underlying comorbidities can influence both the incidence of AF and the electrophysiological characteristics captured by diagnostic tools such as ECGs (see Table 4). Consequently, an AI model trained predominantly on one demographic group may perform sub-optimally or produce biased results when applied to others, potentially exacerbating health disparities [12,13,24].

By systematically collecting and analyzing demographic data during model development and validation, researchers can identify performance gaps and tailor algorithms to improve sensitivity and specificity across all relevant groups. For example, age-related changes in cardiac conduction or sex-specific differences in AF manifestation may require the AI to incorporate different features or thresholds for accurate detection [24,26]. Similarly, ethnic and racial diversity in training datasets ensures that AI tools are robust against population heterogeneity and can provide equitable diagnostic accuracy [25].

## 6. Economic Analysis

The PULsE-AI trial assessed the cost-effectiveness of using a machine learning risk prediction algorithm alongside diagnostic testing to identify undiagnosed AF in primary care settings. Conducted across six general practices in England from June 2019 to February 2021, the study estimated that implementing this screening strategy could result in 45,493 new AF diagnoses among 3.3 million high-risk individuals. This approach was associated with an incremental cost-effectiveness ratio (ICER) of GBP 3994 per quality-adjusted life year (QALY) gained, well below the UK’s accepted threshold of GBP 20,000 per QALY, indicating cost-effectiveness [29].

## 7. Ethical Considerations

AI models for AF detection, particularly ones derived from ECG data, such as those developed by Attia et al. [8], Christopoulos et al. [7], and Dupulthys et al. [12], rely on large-scale patient datasets that inherently contain sensitive health information. Ensuring patient privacy and maintaining robust data security are therefore paramount concerns, especially when such models are integrated into wearable devices or remote monitoring tools, as discussed by Gill et al. [11]. Transparent and comprehensive informed consent protocols are essential, particularly when patient data are repurposed for secondary research beyond the initial scope of collection.

Moreover, the complex nature of machine learning models introduces challenges around interpretability. The rationale behind a given diagnosis may be difficult for both clinicians and patients to understand, potentially leading to overreliance on algorithmic outputs without sufficient contextual insight. While some models, such as those proposed by Attia et al. [8], have attempted to improve interpretability through techniques including saliency mapping, these methods still fall short of full transparency. This barrier may erode trust in the clinician–patient relationship and raise significant concerns regarding accountability, especially in the event of a misdiagnosis or inappropriate treatment decision influenced by AI-generated results.

## 8. Future and Perspectives

The future of AI in detecting and managing AF is moving toward using multiple types of data. By combining these different data sources, AI models could offer more precise and personalized risk assessments. Integrating these algorithms directly into automated ECG interpretations could also allow for real-time AF risk detection during patient visits, helping doctors make quicker and more informed decisions. However, to make sure these tools are accurate and do not unintentionally contribute to health disparities, it is essential that they are trained and tested on diverse populations, as highlighted in Table 2 [30].

Some techniques currently under development, such as Poincaré-plot-based methods, offer an innovative way to visualize heart rate variability by plotting each RR interval against its immediate predecessor. This approach captures the dynamic, beat-to-beat structure of cardiac rhythm irregularities and is especially useful when raw ECG waveforms are unavailable, noisy, or difficult to process—conditions common in wearable or resource-limited settings. Increasingly, researchers have transformed these Poincaré plots into two-dimensional grayscale images, enabling CNNs to learn from the spatial and geometric patterns that emerge from RR interval dispersion [31].

Bashar et al. [31] describe in detail how this method is implemented. First, RR intervals are extracted from ECG signals, and each pair of consecutive intervals (RR_n_, RR_n_₊_1_) is used to construct a Poincaré plot (a scatter diagram that captures the variability in the heart rhythm). These plots are then converted into 2D pixel-based grayscale images, where each point represents a beat pair and the overall distribution encodes the rhythm structure. In cases of sinus rhythm, the resulting image typically forms a tight, elliptical cluster, whereas, in atrial fibrillation, the distribution becomes highly dispersed and chaotic.

These image representations are used as direct inputs to CNNs, which are trained to recognize the visual signatures of arrhythmic versus normal rhythms. CNNs can thereby identify complex, nonlinear relationships in R–R interval variability without relying on raw waveform data. This form of feature extraction enables deep learning models to perform accurate classification even when traditional signal processing methods are limited by noise, motion artifacts, or brief ECG segments. As a result, Poincaré-based CNN inputs serve not only as an effective method for rhythm classification but also as a noise-resilient alternative that can enhance AF detection in challenging environments [31].

Moreover, recent advances in clinical AI systems, particularly those focused on AF predictions from ECG data, are increasingly incorporating federated learning and explainable artificial intelligence to address key challenges in data privacy and clinical trust. Federated learning, as explored by Alreshidi et al. [28], enables multiple hospitals or institutions to collaboratively train AI models without sharing sensitive patient data directly—thus preserving privacy while maintaining model accuracy across diverse populations. At the same time, explainable AI techniques such as saliency mapping (used in Attia et al. [8]) are being applied to improve transparency by highlighting the ECG segments that influence the model’s diagnosis. These methods aim to bridge the gap between AI systems and clinical decision making, supporting the more interpretable and ethically aligned deployment of machine learning tools in healthcare settings.

Furthermore, it is of paramount importance that future AI research in AF detection prioritizes the inclusion of comprehensive demographic datasets and implements strategies to optimize model performance accordingly (see Table 4). Optimizing AI models through demographic stratification not only enhances clinical utility but also builds trust among clinicians and patients by reducing the risk of misdiagnosis in vulnerable populations. Moreover, the transparent reporting of demographic analyses is essential for regulatory approval, ethical accountability, and fostering the broader adoption of AI in clinical settings [4,8].

## 9. Conclusions

Based on the literature reviewed, AI is poised to revolutionize the detection, prediction, and management of AF. By leveraging advanced machine learning and deep learning techniques, AI can analyze both traditional and wearable-device ECG data with exceptional sensitivity and specificity, even identifying patients at risk during periods of normal sinus rhythm. This early and accurate detection creates opportunities for proactive interventions, potentially reducing the burden of stroke, heart failure, and other AF-related complications. Moreover, integrating AI into wearable technologies enables continuous, cost-effective monitoring beyond clinical settings, democratizing access to cardiovascular care.

However, despite these promising developments, significant challenges remain. Data biases, variability in ECG quality, a lack of generalizability across diverse populations, and regulatory and ethical concerns must be carefully addressed to ensure safe, equitable deployment. Future efforts should focus on improving AI model transparency, validating performance through prospective clinical trials, and ensuring patient data privacy. Combining AI-driven analysis with expert clinical oversight will be essential to maximizing benefits while mitigating risks. With continued refinement, AI has the potential not only to enhance early AF detection and risk stratification but also to fundamentally reshape preventive cardiology, ultimately leading to better patient outcomes and more efficient healthcare systems.

Moreover, the current literature lacks consistency in methodological rigor, particularly regarding prospective, multicenter clinical trials. Most studies rely on retrospective data or single-center cohorts, which limits the generalizability and clinical applicability of their findings. To ensure that AI tools are safe, effective, and equitable across diverse populations, there is an urgent need for a standardized validation framework. Such a framework should incorporate prospective study designs, multicenter collaborations, diverse patient demographics, and transparent reporting standards. Establishing this foundation will be critical for moving AI technologies from promising research tools to trusted clinical solutions in the fight against atrial fibrillation.

## Figures and Tables

**Table 1:** Summarized timeline of how AI evolved between the years of 2013 and 2023 [4].

Year Range	Key AI Applications in AF	AI Models or Techniques Used
2013–2014	Very limited AI application in AF. Few studies attempted early exploration.	Basic ML technique
2015–2017	Initial use of AI for stroke risk prediction and AF-related complication modeling.	Bayesian models, Support Vector Machines
2018–2019	Major progress in AI-ECG based AF diagnosis and early use of wearable devices for AF detection.	Deep Neural Networks, Convolutional Neural Networks
2020	AI-assisted catheter ablation planning and AF recurrence prediction.	Deep Convolutional Neural Networks, Recurrent Neural Networks
2021	AI applied to predict complications from AF-related stroke and post-treatment outcomes.	Light Gradient-Boosting Machine
2022	Integration of ECG, imaging, and clinical data to improve AF outcome prediction.	Multimodal Fusion Models
2023	Real-time AF monitoring with wearables and AI for personalized medication and treatment adherence.	Deep Learning for drug monitoring; recurrence detection

**Table 2:** The most prominent and studied models/techniques that can be used in AF detection.

Author(s)	Model/Technology	Brief Description
Attia et al. [8]	Deep Neural Network (DNN) + CNN	A supervised DNN trained on ECGs recorded during sinus rhythm to predict the future incidence of AF. The model processes 12-lead ECG data using a convolutional architecture.
Christopoulos et al. [7]	CNN + Support Vector Machine (SVM) hybrid	Combines a convolutional neural network for feature extraction with a SVM for classification, enhancing precision in short ECG segments.
Chen et al. [9]	Deep CNN on wearable ECG	Utilizes a deep convolutional neural network to analyze single-lead ECG signals from wearable devices in real-time for AF detection.
Gill et al. [11]	Smartphone based model (meta-analysis)	Applies self-attention mechanisms to model long-term dependencies in ECG signals, improving accuracy in identifying arrhythmia patterns.
Dupulthys et al. [12]	Stacking Ensemble (CNN, LSTM, RF) + ArNet2	Integrates multiple classifiers—including CNN, Long Short-Term Memory (LSTM), and Random Forest (RF)—into an ensemble to enhance generalization and robustness.

**Table 3:** Summary and comparison of the performance of various AI models and technologies used for AF detection.

Author(s)	Model/Technology	Sensitivity	Specificity	Accuracy
Attia et al. [8]	DNN + CNN	82.30%	83.40%	83.30%
Chen et al. [9]	Deep CNN on wearable ECG/PPG	80.00%	96.81%	90.52%
Gill et al. [11] (multiple studies)	PPG smartphone	94.00% (pooled)	97.00% (pooled)	61.00–99.00% (range)
Alreshidi et al. [28]	AI ECG + Federated Learning (LTSM, SVM)	96.60%	81.80%	94.20%
Mol et al. [23]	PPG-AI based algorithm	98.10%	98.10%	N/R
Biton et al. [24]	Deep learning CNN	Female: 92.00% Male: 86.00%	Female: 98.00% Male: 97.00%	N/R

**Table 4:** Most important results of the demographic analysis conducted in the various studies analyzed in this review.

Study	AI model Used	Cohort/Sample Size	Demographic Subgroups Reported	Demographic Finding
Biton et al. [24]	Deep learning CNN	Multi cohort US, Japan and others	Age, sex, geographic location	Model performance consistent across age groups and sexes; some geographical variation noted, with slightly reduced accuracy in older populations.
Attia et al. [8]	Deep Learning CNN (end to end)	180,922 patients with 649,931 ECGs	Age, sex	Slightly higher sensitivity in younger patients; performance consistent between males and females.
Christopoulos et al. [7]	Deep neuronal network	1936 patients	Age, sex, race	Model tested across racial/ethnic subgroups; minor differences in specificity but maintained overall accuracy.
Krivoshi et al. [13]	Support vector machine and CNN hybrid	80 patients	Age, sex	Younger patients showed better detection rates; sex differences not significant.
Gill et al. [11]	Multiple AI models (meta-analysis)	11,404 patients	Age, sex	AI models maintained high accuracy across sexes; some decline in accuracy in elderly cohorts noted in PPG-based devices.
Popat et al. [25]	Ensemble methods such as stacking	109 studies (precise numbers unavailable)	Age, sex, race	Model performance consistent across races; slightly better sensitivity in females; age-related performance variation highlighted.
Isaksen et al. [5]	CNN and ensemble methods	Multiple study review (no precise cohort numbers present)	Age, sex, race	Review noted limited demographic reporting in original studies; calls for more inclusive datasets to improve generalizability.
Dupulthys et al. [12]	Single-lead ECG AI model with risk factors (Stacking method and ArNet2 model)	173,537 ECGs from 68,880 patients (used to train model) 13,479 ECGs tested	Sex	Sex distribution was balanced, and the model maintained consistent predictive performance across sexes, indicating demographic robustness in detection accuracy.

## Abbreviations

The following abbreviations are used in this manuscript:

AI	Artificial Intelligence
AF	Atrial fibrillation
ECG	Electrocardiograph
ML	Machine learning
CNNs	Convolutional Neural Networks
PPG	Photoplethysmogram
AI-ECG	Artificial Intelligence-Enabled Electrocardiogram
AUC	Area under the curve
PPV	Positive predictive value
ICER	Incremental cost-effectiveness ratio
QALY	Quality-adjusted life year
